# Diagnostic value of dual-source CT dual-energy technology for assessing differentiation degree and serosal invasion in colorectal cancer: A retrospective study

**DOI:** 10.1097/MD.0000000000049556

**Published:** 2026-07-03

**Authors:** Jingduo Wang, Aibo Wang, Xuehua Qin, Xiaoyuan Li, Chunmao Cui, Ling Liu

**Affiliations:** a Department of Radiology, No. 92493 Unit Hospital of the Chinese People’s Liberation Army, Huludao, Liaoning, China; b Department of Radiology, The First Affiliated Hospital of Dali University, Dali University, Dali, Yunnan, China.

**Keywords:** colorectal cancer (CRC), differentiation degree, dual-source CT (DSCT), serosal invasion

## Abstract

To evaluate the diagnostic efficacy of dual-source CT (DSCT) dual-energy technology in determining the differentiation degree and presence of serosal invasion in colorectal cancer (CRC), using normalized iodine concentration (NIC), electron density, and effective atomic number (*Z*) as diagnostic markers. A retrospective analysis was conducted on 102 patients clinically diagnosed and pathologically confirmed with CRC. Dual-energy images from both arterial and venous phases were obtained using third-generation DSCT. NIC values were computed. The study analyzed the diagnostic value of DSCT dual-energy technology, utilizing multiple parameters, in assessing the differentiation degree of CRC and the presence of serosal invasion. For differentiating high/moderate differentiation from low differentiation, the AUCs of NIC and *Z* were 0.899 and 0.914 in the arterial phase and 0.865 and 0.891 in the venous phase, respectively. For detecting serosal invasion, the arterial-phase AUCs of NIC and *Z* were 0.963 and 0.944, respectively, and the venous-phase AUC of NIC was 0.962. *ρ* showed no significant diagnostic value in these comparisons. DSCT dual-energy technology, through NIC and effective atomic number, offers significant diagnostic value for evaluating both the differentiation degree and serosal invasion in CRC, providing a novel approach for preoperative assessment.

## 1. Introduction

Colorectal cancer (CRC) is one of the most common malignancies of the digestive tract and is a major cause of cancer-related morbidity and mortality worldwide. In China, CRC also remains among the leading cancers in both incidence and mortality.^[1,2]^

The vast majority of CRC cases are adenocarcinomas, often presenting as villous-tubular adenocarcinomas. Pathologically, these can be classified into 4 differentiation grades: high, moderate, low, and undifferentiated.^[3]^ According to the AJCC’s ninth edition staging system for CRC, T-stage T4a is characterized by tumor penetration of the visceral peritoneum, whereas T4b indicates direct invasion or adhesion to other organs or structures. Therefore, tumors that invade the serosa are categorized as T4a, whereas those infiltrating the muscularis propria without serosal invasion are classified as T3.

Factors such as diet, obesity, lack of physical activity, smoking and moderate-to-heavy alcohol consumption can increase the risk of developing CRC. On the contrary, it has been reported that higher intakes of dietary fiber, leafy vegetables, folic acid and calcium can prevent CRC.^[4]^

Multi-detector computed tomography (MDCT) is widely used for preoperative evaluation because it provides rapid acquisition and high spatial resolution. However, conventional MDCT mainly relies on morphologic criteria, such as tumor size, bowel-wall thickening, and lymph-node enlargement, which may be insufficient for assessing tumor differentiation or biological heterogeneity.^[5]^

Dual-energy CT (DECT) technology substantially mitigates the challenges posed by the reliance on a single parameter (CT number) in MDCT during clinical diagnostic and therapeutic processes. Through post-processing, DECT enables the acquisition of virtual non-contrast images (VNC), iodine maps, and dual-energy indices (DEI), as well as images representing electron density and effective atomic number (*ρ*/*Z*). This technique allows for the direct measurement of lesion iodine concentration (IC), *ρ* values, and *Z* values. Thus, quantitative analysis of the extent of tumor infiltration and its histological types can be performed.^[6]^ Third-generation DSCT further improves scanning speed, image quality, quantitative parameter acquisition, and radiation-dose efficiency.

DSCT is now employed in the preoperative grading and staging of gastric cancer,^[7,8]^ with studies indicating correlations between DSCT’s quantitative parameters, such as normalized iodine concentration (NIC), spectral HU curve slope, and effective atomic number, with T staging.^[9,10]^ However, there is a paucity of research, both domestically and internationally, on the differentiation degree of CRC. Therefore, this study investigated the value of NIC, *ρ*, and *Z* for preoperative assessment of CRC differentiation degree and serosal invasion.

## 2. Materials and methods

### 2.1. Data collection

A retrospective analysis was conducted on 102 patients diagnosed with CRC confirmed by surgery and pathology from December 2018 to January 2021, who underwent DSCT scans. Inclusion criteria included: patients with a pathological diagnosis of CRC and complete clinical, pathological, and imaging data; patients diagnosed for the first time without any prior intervention treatments; and patients able to undergo DSCT enhanced scanning without contraindications. Exclusion criteria included: patients who had received clinical interventions such as radiotherapy, chemotherapy, molecular targeted therapy, and preoperative neoadjuvant chemotherapy; patients contraindicated for DSCT examination and those allergic to iodine-based contrast agents; patients whose post-diagnosis pathological results could not provide CRC grading and staging; patients with recurrent CRC; and patients whose DSCT imaging data and surgical timing exceeded 1 month.

This study was approved by the ethics committee of Dali University (Approval No. DFY20251105001). The requirement for written informed consent was waived because of the retrospective design and use of de-identified data. The ethics approval document is provided as Supplementary Material 1.

### 2.2. Examination methods

The third-generation DSCT scanner (SOMATOM Force, Siemens, Germany) was used to perform a full abdomen dual-energy scan. The patient was positioned supine, head-first, from the top of the diaphragm to the inferior margin of the pubic symphysis. Scanning parameters were: tube voltage 90 KVp, tube current 220 mA s, gantry rotation time 0.5 seconds, pitch 0.6, collimator specifications 192 mm × 0.6 mm, scan slice thickness and inter-slice spacing 5 mm. Iodixanol (300 mg I/mL) was injected through the antecubital vein at 1.5 mL/kg and 3 mL/s using a dual-barrel high-pressure injector. After reaching a threshold of 100 HU in the abdominal aorta, an automatic arterial phase scan was performed after a 7-second delay, followed by a venous phase scan after a 25-second delay. Scanning parameters: the tube voltages for the 2 tubes were 100 kV and Sn150 kV respectively, with maximum tube currents of 180 A s and 90 A s. Upon completion of the scan, the CT images were transferred to the picture archiving and communication system (PACS) and the post-processing workstation.

### 2.3. Image post-processing

Utilizing the Siemens syngo MMWP VE36A workstation, the “Liver VNC” mode was selected to import thin-layer images from both arterial and venous phases. Tumor sites were identified by 2 experienced diagnostic radiologists (reader 1 and reader 2, possessing 8 and 15 years of experience, respectively), who were blinded to clinical and pathological data. For the delineation of the region of interest (ROI) from which ICs were derived, the axial slice that displayed the largest cross-sectional area of the tumor was selected for analysis. On this slice, an ROI was meticulously placed on the most homogeneously enhanced solid portion of the tumor. The size of each ROI was approximately 40 to 60 mm^2^, explicitly avoiding areas of necrosis, calcification, and intraluminal air by referencing conventional CT images. Measurements were repeated 3 times on the same layer, and the average value was recorded. For the perienteric fat space, a single 10 mm^2^ ROI was positioned at a distance of 1 mm from the intestinal wall adjacent to the lesion, carefully avoiding visible vessels. The *ρ*/*Z* mode was then used to obtain *ρ* and *Z* values from the tumor and perienteric fat ROIs. NIC was calculated as the ratio of lesion IC to arterial IC on the same slice in each phase.

### 2.4. Statistical analysis

The intraclass correlation coefficient (ICC), interpreted as follows: 0.00 to 0.20 indicates poor correlation; 0.21 to 0.40, fair correlation; 0.41 to 0.60, moderate correlation; 0.61 to 0.80, good correlation; 0.81 to 1.00, excellent correlation, was used to assess intraobserver reliability and interobserver agreement. Continuous variables were expressed as mean ± standard deviation (SD). The Shapiro–Wilk test was used to assess normality. For comparisons among the 3 differentiation groups (high, moderate, and low), 1-way analysis of variance (ANOVA) was used for normally distributed variables; otherwise, the Kruskal–Wallis *H* test was used, followed by Bonferroni-corrected post hoc pairwise comparisons when appropriate. For the 2 serosal-invasion groups, independent-samples *t* tests were used for normally distributed variables, and the Mann–Whitney *U* test was used for non-normally distributed variables. Receiver operating characteristic (ROC) curves were plotted to assess diagnostic efficacy. The optimal cutoff values were determined by maximizing the Youden index (sensitivity + specificity − 1). Pairwise comparisons of the areas under the ROC curves (AUCs) were performed using the DeLong test.

This was an exploratory retrospective study based on a convenience sample. A formal a priori sample size calculation was not performed because preliminary data for these specific DSCT quantitative parameters were unavailable. Nevertheless, the sample size of this study (n = 102) is comparable with previous spectral CT studies evaluating CRC histologic grade or stage, which enrolled 131 and 165 patients.^[9,10]^

All statistical analyses were performed using SPSS version 25.0 (IBM Corp), and a *P* value of <.05 was considered statistically significant. This study had no missing data.

## 3. Results

### 3.1. Clinical data of patients

The study included 102 cases of CRC patients, comprising 54 males and 48 females. The age range was from 40 to 88 years, with a mean age of 63.1 ± 9.1 years and a median age of 63 years. The tumor location was in the rectum for 75 cases and in the colon for 27 cases. According to surgical pathology, 17 tumors were highly differentiated, 63 were moderately differentiated, and 22 were poorly differentiated. Serosal invasion was present in 69 patients and absent in 33 patients. No significant differences in age distribution were observed among the study groups.

### 3.2. Reproducibility of measurements

Interobserver and intraobserver reproducibility for NIC, *ρ*, and *Z* in both arterial and venous phases were assessed using the intraclass correlation coefficient (ICC). As shown in Table 1, all ICC values exceeded 0.85, indicating excellent reproducibility for all measurements across both phases.

**Table 1:** Intra- and interobserver reproducibility for NIC, *ρ*, and *Z* values measurements.

Parameter	Phase	Intraobserver reliability (ICC, 95% CI)	Interobserver agreement (ICC, 95% CI)
NIC	Arterial	0.94 (0.90–0.97)	0.91 (0.86–0.95)
	Venous	0.93 (0.89–0.96)	0.90 (0.85–0.94)
*ρ*	Arterial	0.90 (0.85–0.94)	0.88 (0.82–0.93)
	Venous	0.89 (0.84–0.93)	0.87 (0.81–0.92)
*Z*	Arterial	0.95 (0.92–0.97)	0.93 (0.89–0.96)
	Venous	0.94 (0.90–0.97)	0.92 (0.88–0.95)

CI = confidence interval, ICC = intra-class correlation coefficient, NIC = normalized iodine concentration.

### 3.3. Diagnostic analysis of differentiation degree of CRC during arterial and venous phases

**Figure 1.**
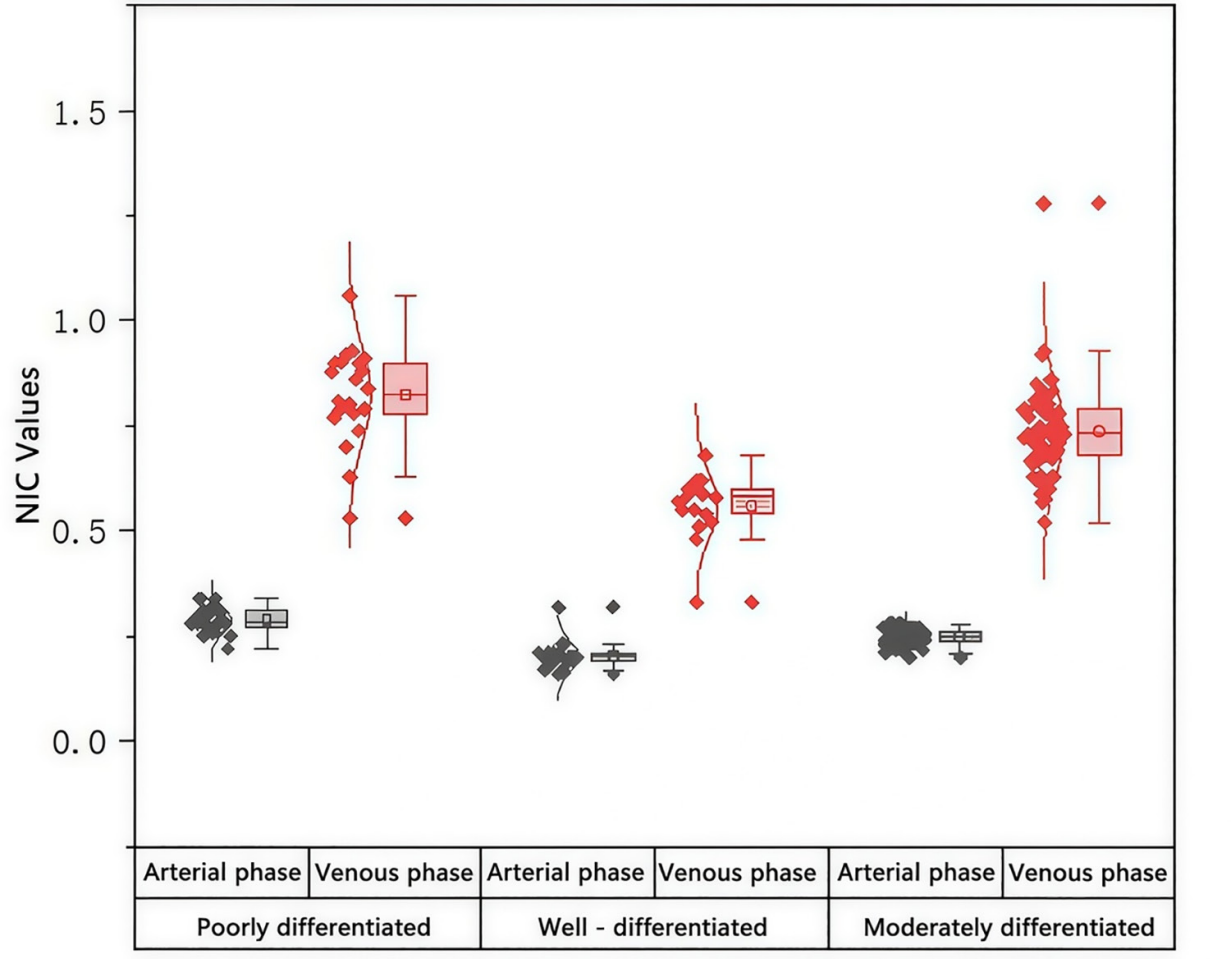
Distribution of NIC values according to CRC differentiation degree during arterial and venous phases (scatter plot + normal distribution curve + box plot). AP = arterial phase, CRC = colorectal cancer, NIC = normalized iodine concentration, VP = venous phase.

**Figure 2.**
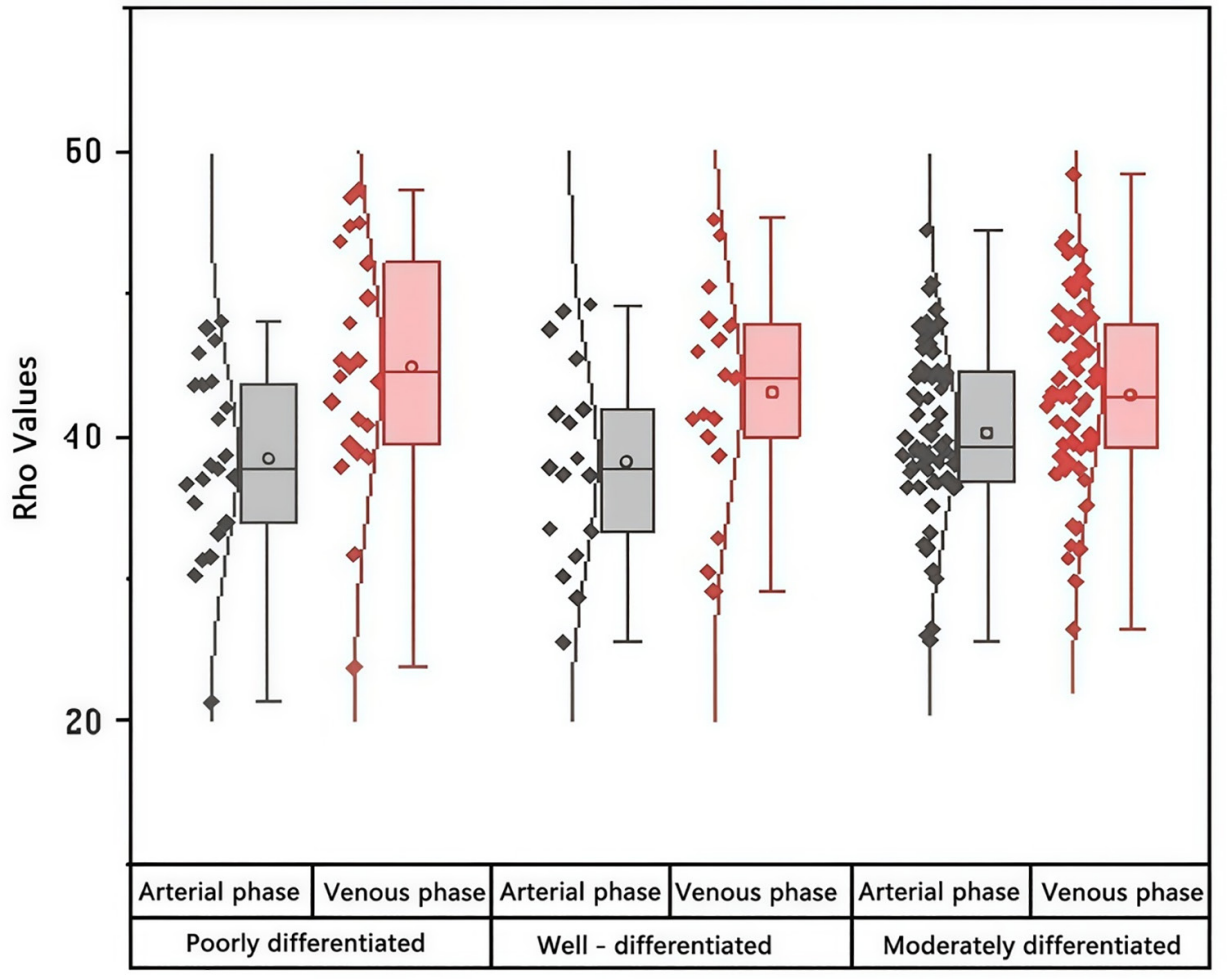
Distribution of *ρ* values in CRC lesions with different degrees of differentiation during arterial and venous phases. CRC = colorectal cancer.

**Figure 3.**
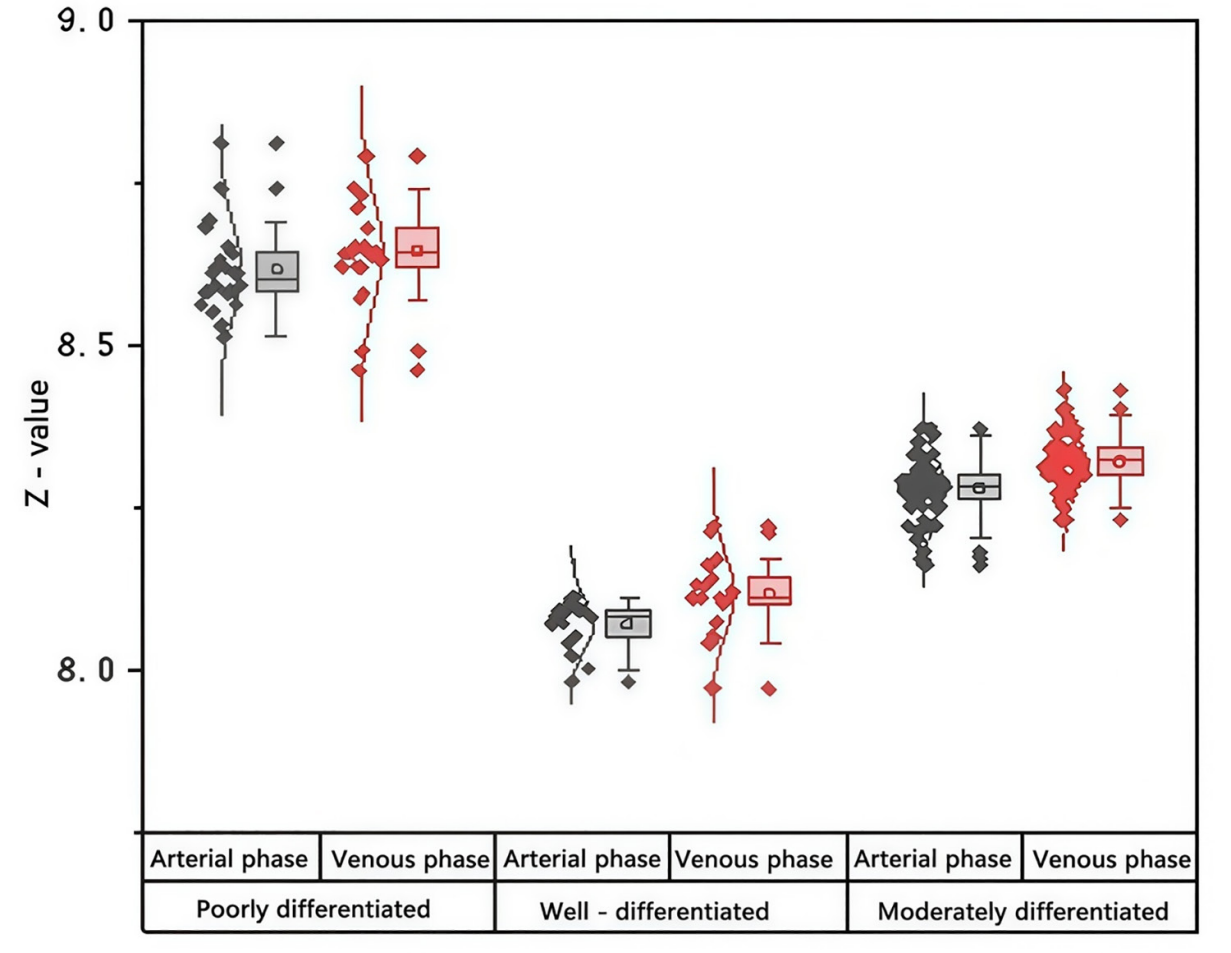
Distribution of *Z* values according to CRC differentiation degree during arterial and venous phases. CRC = colorectal cancer.

During the arterial phase, parameters such as NIC, *ρ*, and *Z* for the 3 groups (high, moderate, and low differentiation) conformed to a normal distribution and met the assumptions of homogeneity of variance (Figs. 1–3), and were thus evaluated using 1-way ANOVA (Table 2). The statistical outcomes indicated significant differences in NIC and *Z* among the 3 groups (*P* < .05), with post hoc Bonferroni-corrected pairwise comparisons also showing significant differences (*P* < .05); however, *ρ* showed no significant differences across the groups (*P* > .05).

**Table 2:** Comparison of NIC, *ρ*, and *Z* values during the arterial phase among CRCs of different differentiation degrees.

Grouping	NIC	*ρ*	*Z*
Group1	0.20 ± 0.04	38.18 ± 7.11	8.07 ± 0.04
Group2	0.25 ± 0.02	40.18 ± 6.05	8.28 ± 0.05
Group3	0.29 ± 0.03	38.38 ± 6.62	8.61 ± 0.07
*F* value	57.677	1.064	590.46
*P* value	.000*	.349	.000*

Group 1: high differentiation, group 2: moderate differentiation, group 3: low differentiation; Data are presented as mean ± standard deviation.

CRC = colorectal cancer, NIC = normalized iodine concentration.

* *P* < .05 was considered statistically significant.

During the venous phase, the parameters (NIC, *ρ*, and *Z*) for the 3 groups also conformed to a normal distribution and satisfied the criteria for homogeneity of variance (Figs. 1–3), leading to analysis using 1-way ANOVA (Table 3). There were statistically significant differences in NIC and *Z* among the 3 groups (*P* < .05). Post hoc Bonferroni-corrected pairwise comparisons among the groups also showed statistically significant differences (*P* < .05); the *ρ* difference in the 1-way ANOVA among the 3 groups was not statistically significant (*P* > .05).

**Table 3:** Comparison of NIC, *ρ*, and *Z* values during the venous phase among CRCs of different differentiation degrees.

Grouping	NIC	*ρ*	Z
Group1	0.56 ± 0.08	43.06 ± 7.46	8.11 ± 0.06
Group2	0.74 ± 0.11	42.95 ± 6.46	8.32 ± 0.04
Group3	0.82 ± 0.11	44.79 ± 8.34	8.64 ± 0.08
*F* value	31.254	0.569	473.891
*P* value	.000*	.568	.000*

Data are presented as mean ± standard deviation.

CRC = colorectal cancer, NIC = normalized iodine concentration.

* *P* < .05 was considered statistically significant.

### 3.4. Diagnostic efficacy of different parameters during the arterial and venous phases for assessing the differentiation degree of CRC

This study categorized the differentiation degrees of CRC into high/moderate differentiation and low differentiation groups. Statistically significant parameters were further analyzed using ROC curves.

**Figure 4.**
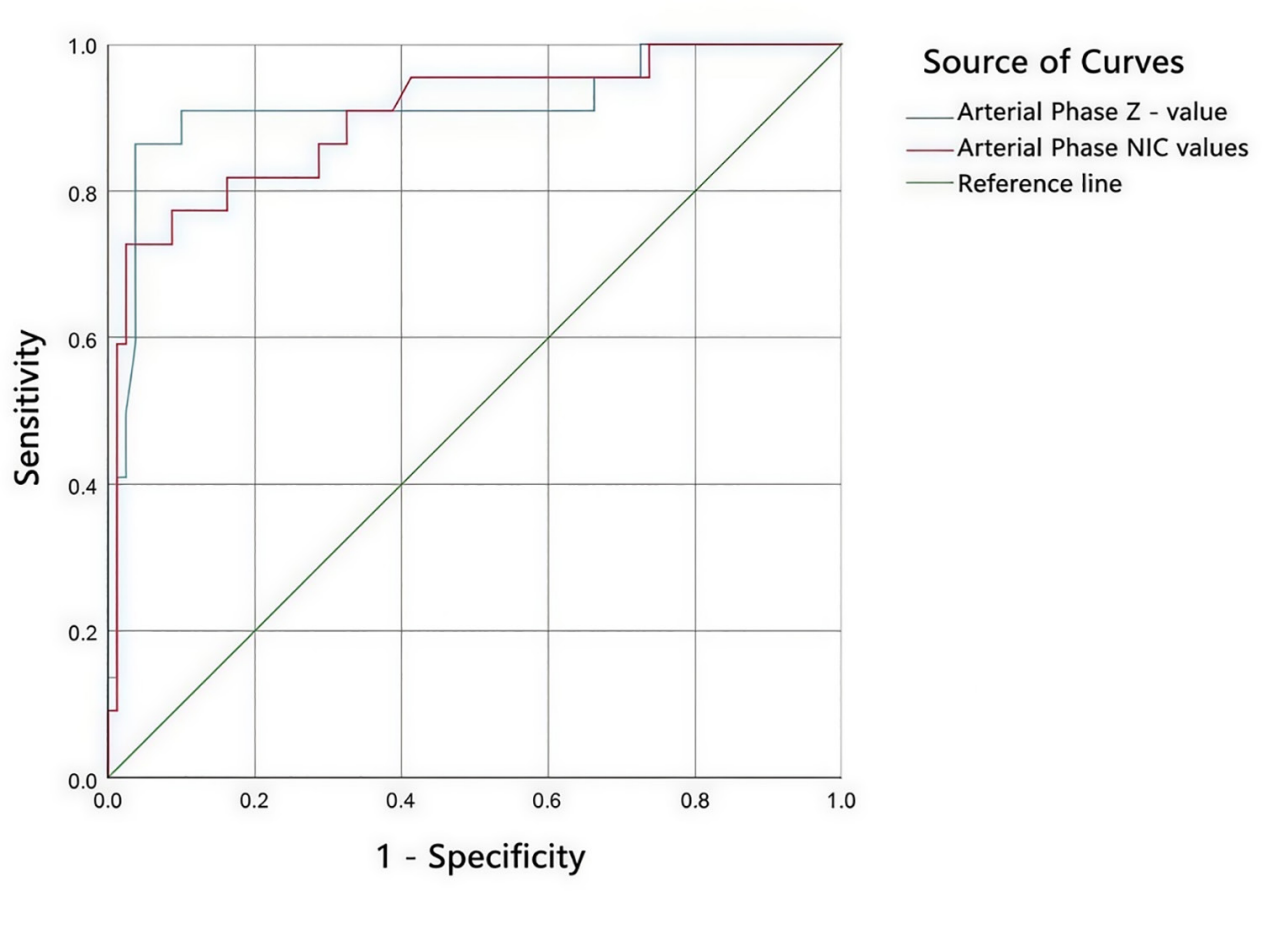
ROC curves for the assessment of CRC differentiation (high/moderate vs low) using *Z* and NIC values during the arterial phase. CRC = colorectal cancer, NIC = normalized iodine concentration, ROC = receiver operating characteristic.

During the arterial phase, the AUC for *Z* and NIC in assessing the differentiation degree of CRC were 0.914 and 0.899, respectively (Fig. 4). The sensitivity was recorded at 86.4% for both *Z* and NIC, while the specificity was 96.3% for *Z* and 71.2% for NIC. The positive predictive values were 86.3% for *Z* and 42.2% for NIC, and the negative predictive values were 96.2% for *Z* and 94.7% for NIC. The optimal cutoff values were 8.511 for *Z* and 0.262 mg/mL for NIC (Table 4). DeLong testing showed no significant difference between the AUCs (*P* > .05).

**Table 4:** Diagnostic efficacy of *Z* and NIC values for assessing CRC differentiation (high/moderate vs low) during the arterial phase.

Quantitative parameter	AUC	95% confidence interval		*P* value	Cutoff value	Sensitivity (Se) %	Specificity (Sp) %	PPV (%)	NPV (%)
		Upper limit	Lower limit						
*Z*	0.914	0.999	0.828	<.001	8.51	86.4	96.3	86.3	96.2
NIC	0.899	0.980	0.818	<.001	0.26 mg/mL	86.4	71.2	42.2	94.7

AUC = area under the curve, CRC = colorectal cancer, NIC = normalized iodine concentration, NPV = negative predictive values, PPV = positive predictive values.

**Figure 5.**
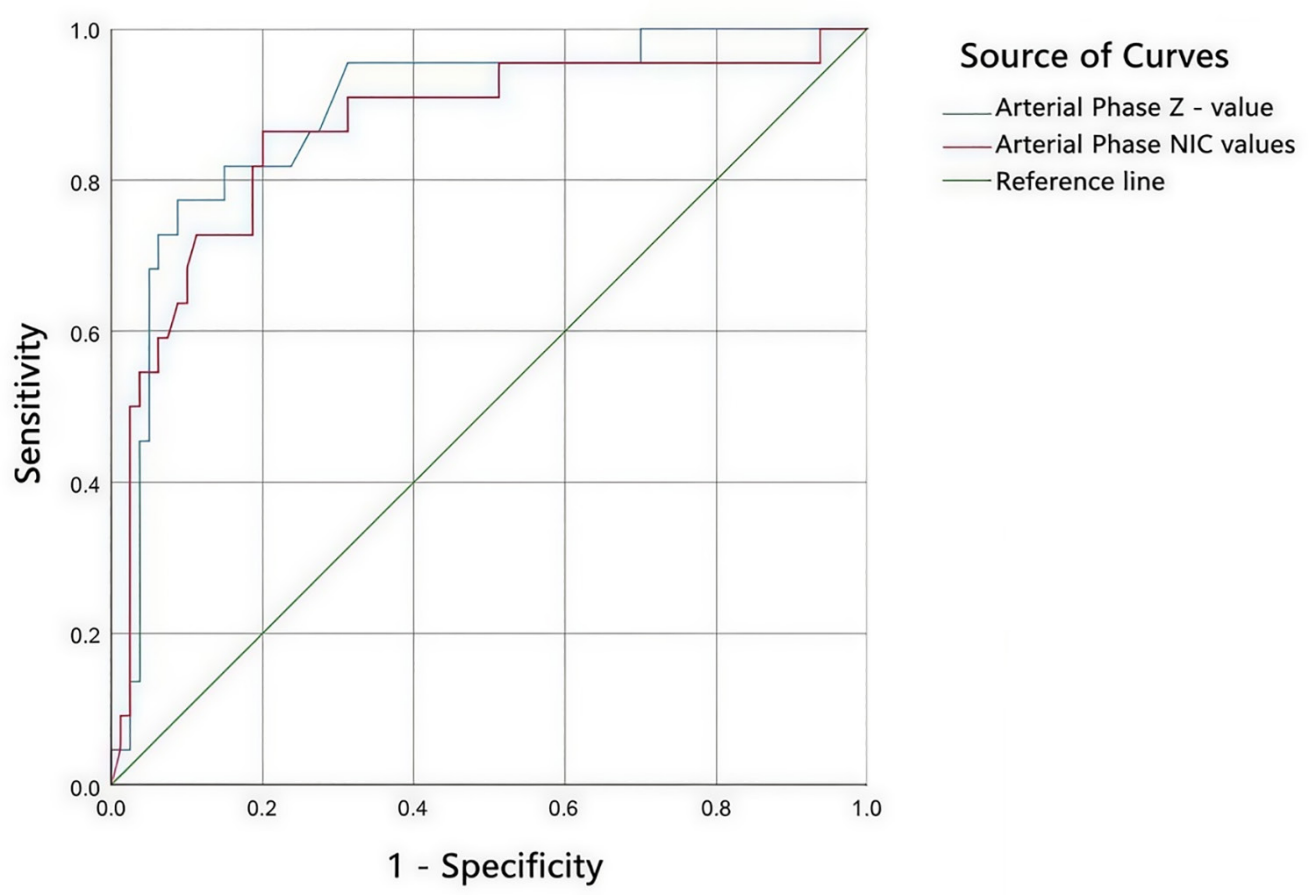
ROC curves for the assessment of CRC differentiation (high/moderate vs low) using *Z* and NIC values during the venous phase. CRC = colorectal cancer, NIC = normalized iodine concentration, ROC = receiver operating characteristic.

During the venous phase, the AUCs for *Z* and NIC in evaluating the differentiation degree of CRC were 0.891 and 0.865, respectively (Fig. 5). The sensitivity was 77.3% for *Z* and 86.4% for NIC; specificity was 91.2% for *Z* and 80.0% for NIC. Positive predictive values were 70.8% for *Z* and 51.3% for NIC, and negative predictive values were 92.3% for *Z* and 95.4% for NIC. The optimal cutoff values were 8.445 for *Z* and 0.777 mg/mL for NIC (Table 5). DeLong testing showed no significant difference between the AUCs (*P* > .05).

**Table 5:** Diagnostic efficacy of *Z* and NIC values for assessing CRC differentiation (high/moderate vs low) during the venous phase.

Quantitative parameter	AUC	95% confidence interval		*P* value	Cutoff value	Sensitivity (Se) %	Specificity (Sp) %	PPV (%)	NPV (%)
		Upper limit	Lower limit						
*Z*	0.891	0.968	0.814	<.001	8.45	77.3	91.2	70.8	92.3
NIC	0.865	0.962	0.769	<.001	0.78 mg/mL	86.4	80.0	51.3	95.4

AUC = area under the curve, CRC = colorectal cancer, NIC = normalized iodine concentration, NPV = negative predictive values, PPV = positive predictive values.

### 3.5. Analysis of diagnostic parameters for serosal invasion in CRC during arterial and venous phases

**Figure 6.**
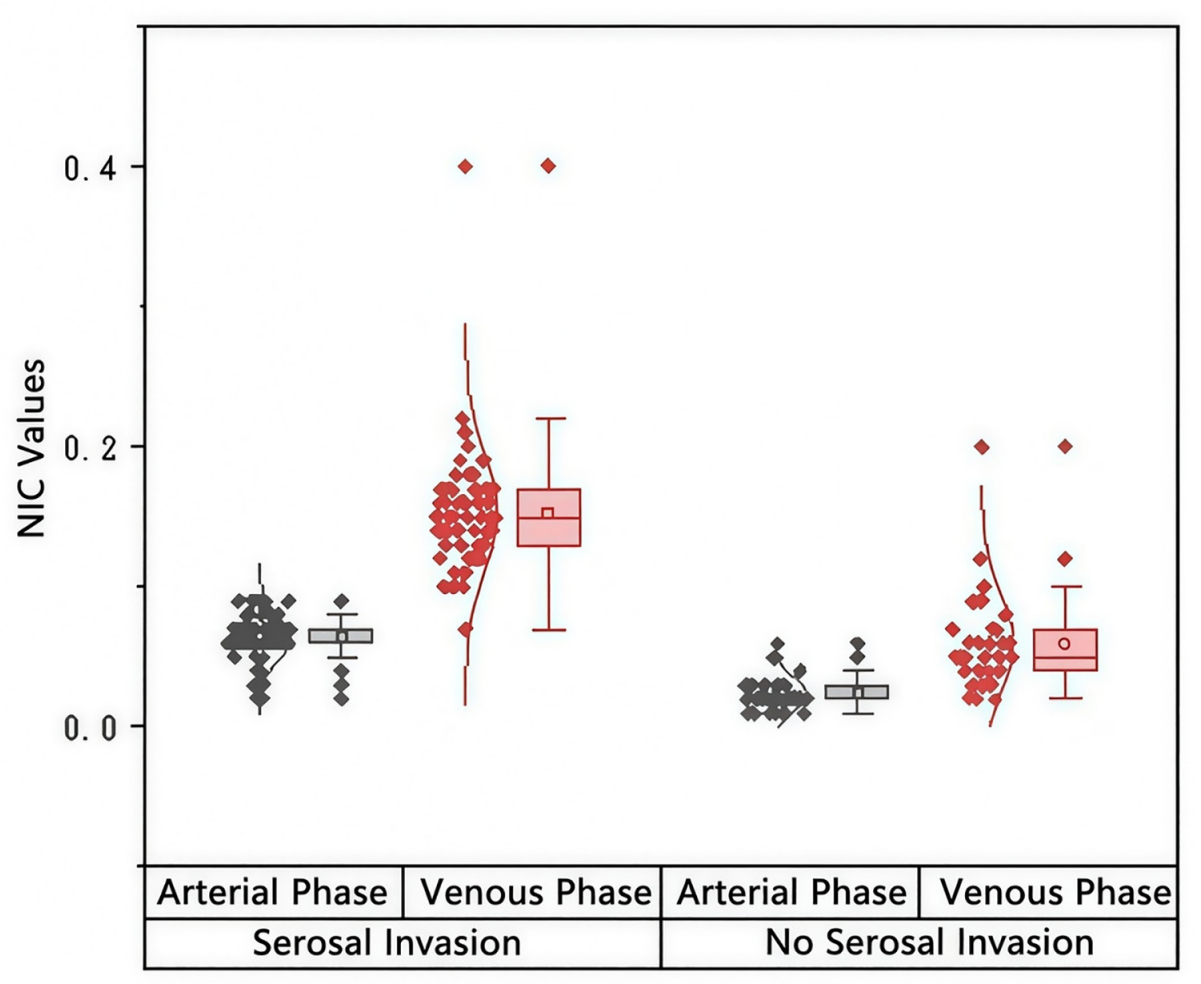
Distribution of NIC values for CRC groups with and without serosal invasion during arterial and venous phases. CRC = colorectal cancer, NIC = normalized iodine concentration.

**Figure 7.**
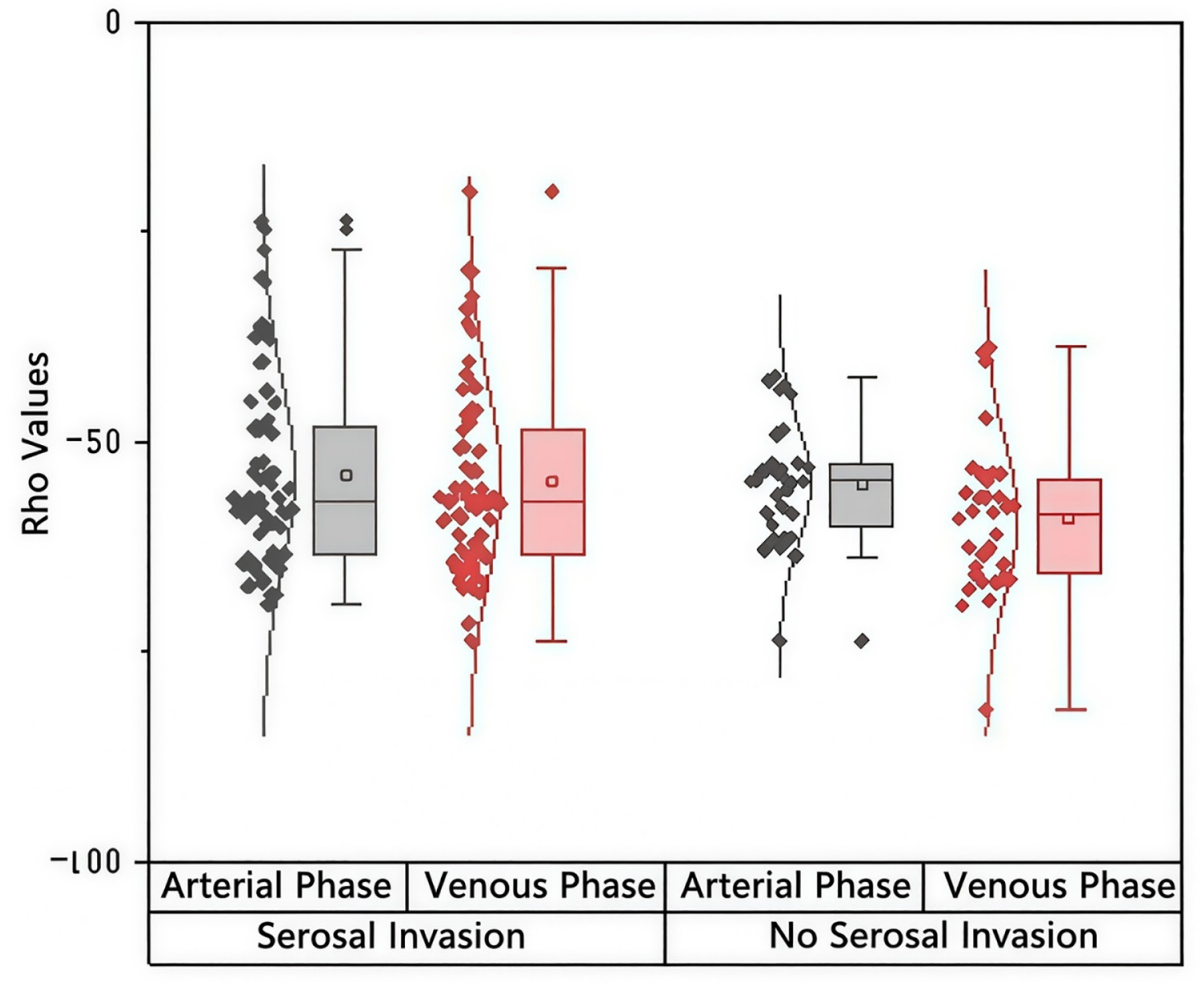
Distribution of *ρ* values for CRC groups with and without serosal invasion during arterial and venous phases. CRC = colorectal cancer.

**Figure 8.**
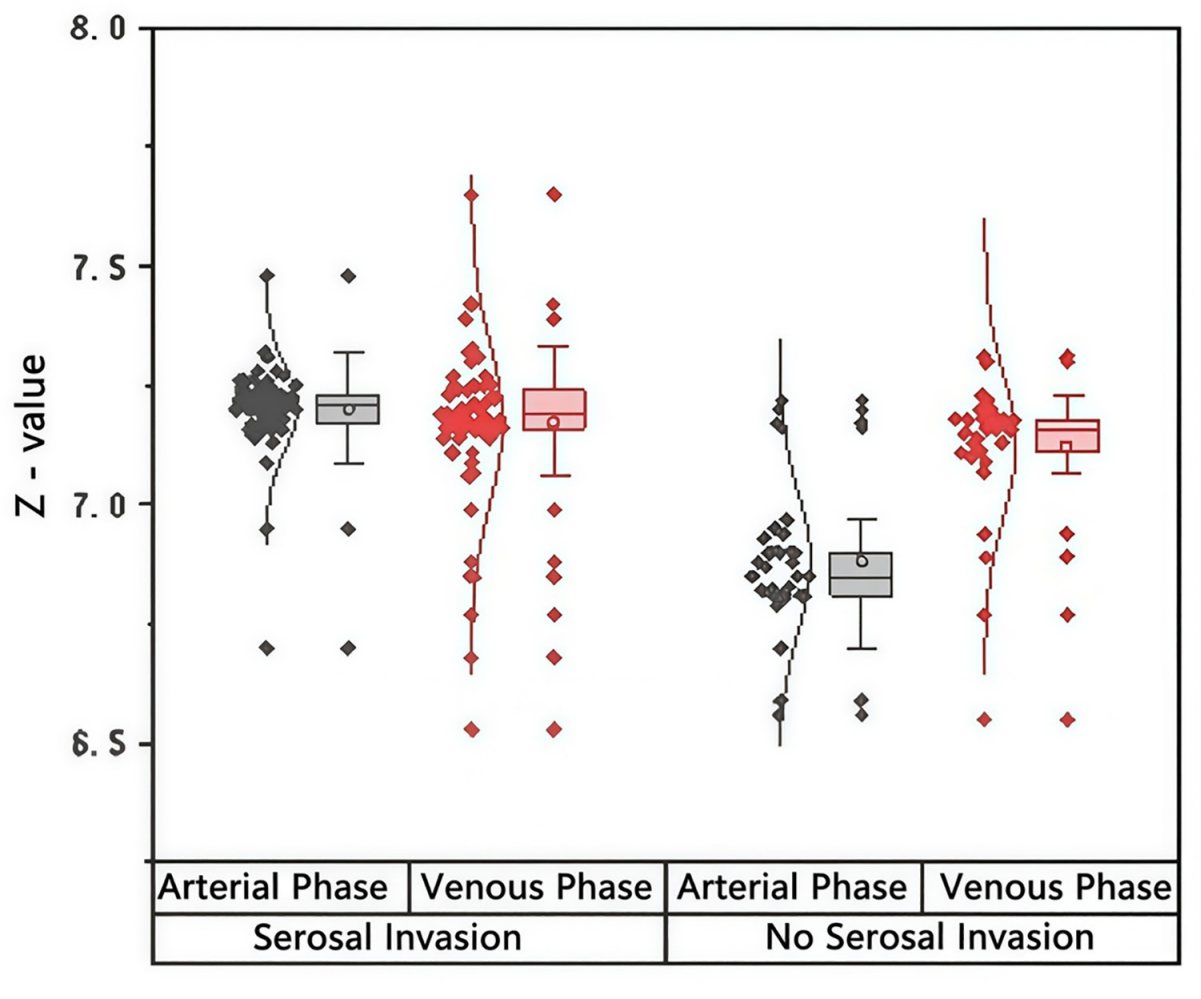
Distribution of *Z* values for CRC groups with and without serosal invasion during arterial and venous phases. CRC = colorectal cancer.

**Figure 9.**
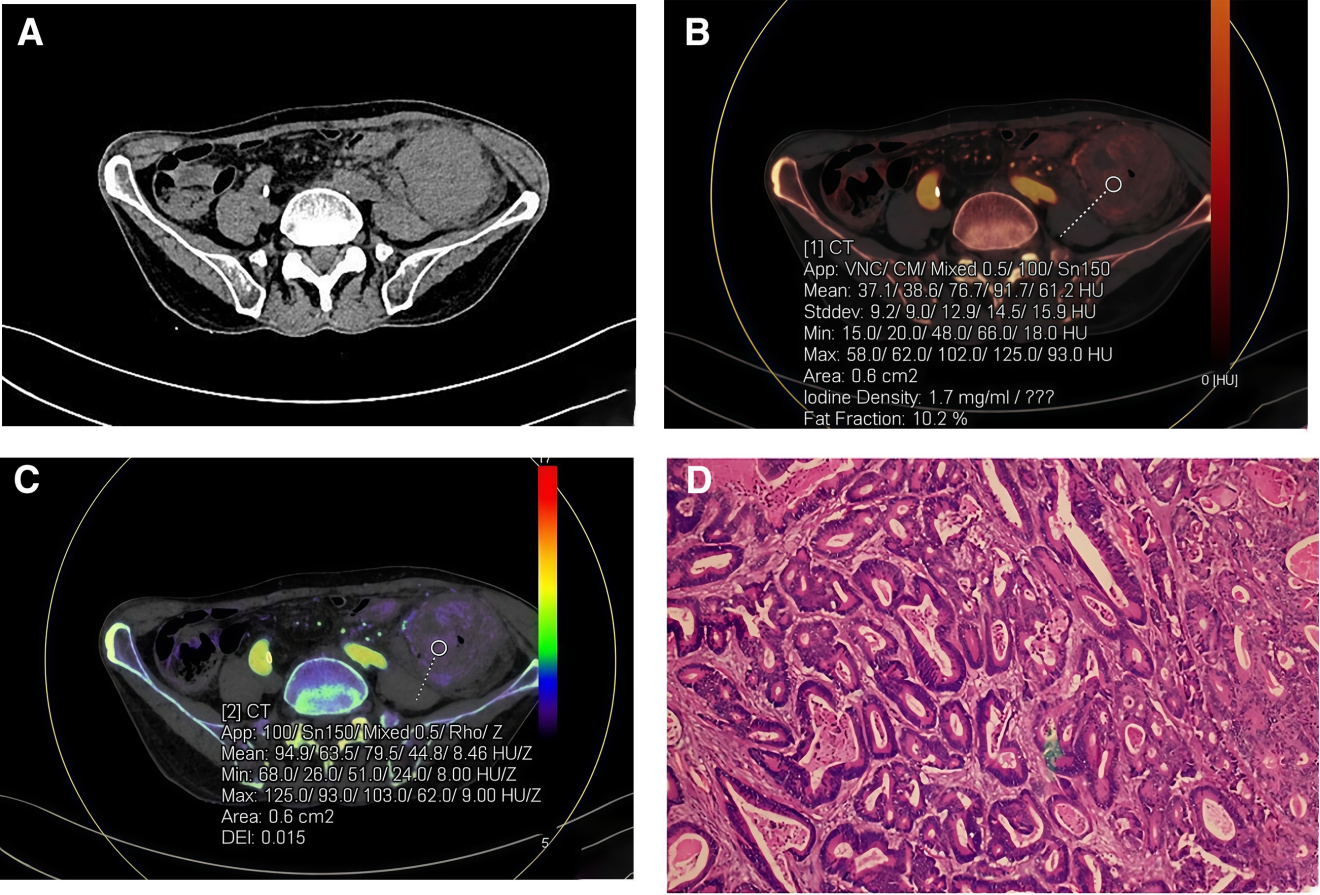
Female, 66 years old, diagnosed with well-differentiated rectal adenocarcinoma. (A) Unenhanced CT image shows irregular thickening of the rectal wall and narrowing of the lumen. (B) The iodine map from the arterial phase shows an iodine concentration of 1.7 mg/mL in arterial phase lesions. (C) The *ρ*/Z map from the arterial phase shows the electron density and effective atomic number diagram, indicating an electron density of 44.5 HU and an effective atomic number of 8.46 at the lesion site during the arterial phase. (D) Photomicrograph (HE ×100) depicts a well-differentiated rectal adenocarcinoma. CT = computed tomography, VNC = virtual non-contrast.

**Figure 10.**
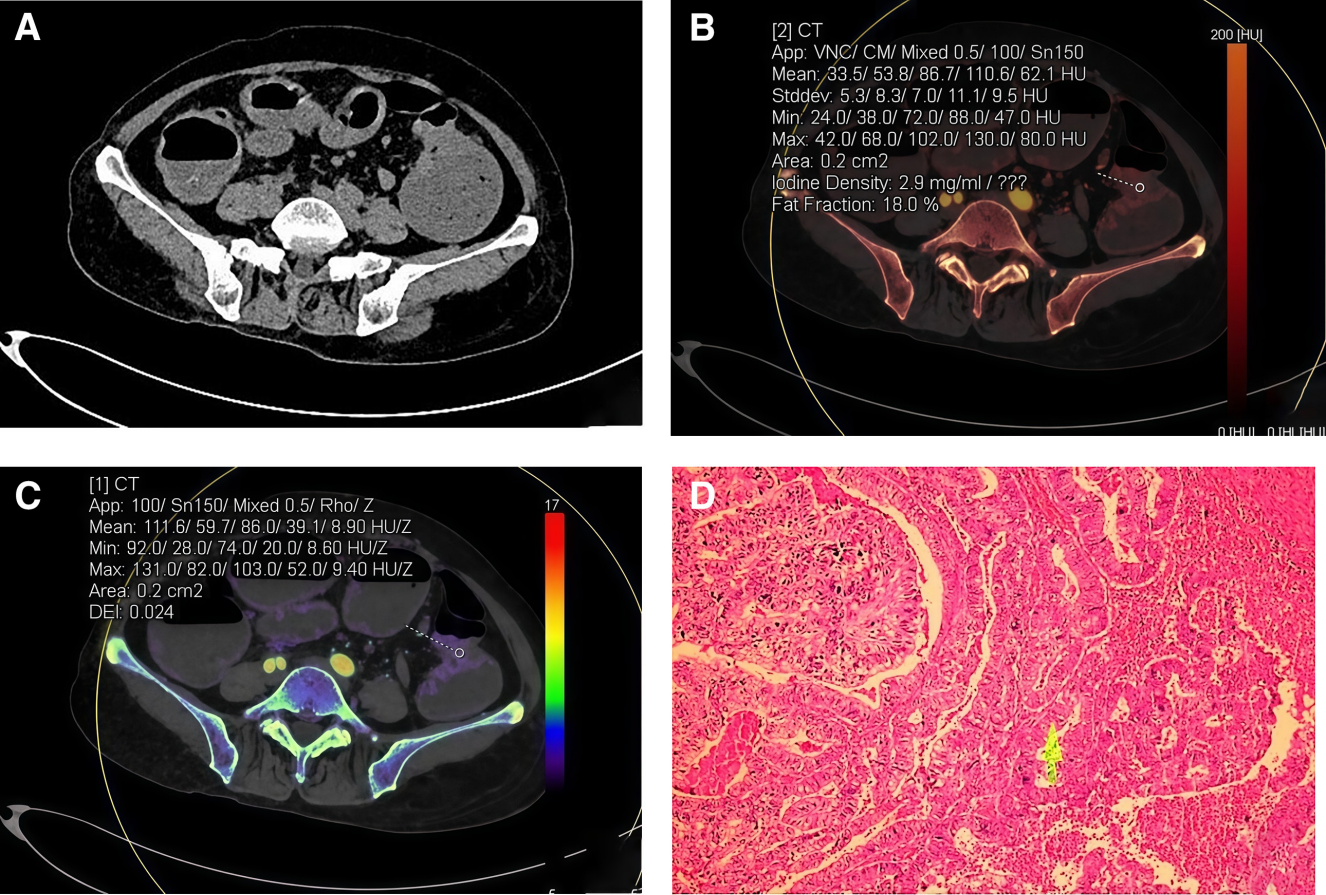
Female, 53 years old, with moderately differentiated rectal adenocarcinoma. (A) Unenhanced CT image shows irregular thickening of the rectal wall and narrowing of the lumen. (B) The iodine map from the arterial phase shows an iodine concentration of 2.9 mg/mL in arterial phase lesions. (C) The *ρ*/*Z* map from the arterial phase shows the electron density and effective atomic number diagram, showing an electron density of 39.1 HU and an effective atomic number of 8.90 at the lesion site during the arterial phase. (D) Photomicrograph (HE ×100) illustrates a moderately differentiated rectal adenocarcinoma. CT = computed tomography, VNC = virtual non-contrast.

This study analyzed the diagnostic relevance of various parameters (NIC, *ρ*, and *Z*) during the arterial and venous phases for detecting serosal invasion in CRC. In the arterial phase, NIC, *ρ*, and *Z* were normally distributed in both groups (Figs. 6–8). Representative DSCT images and corresponding pathological results for well-differentiated, moderately differentiated, and poorly differentiated CRC are shown in Figs. 9A–D, 10A–D, and11A–D, respectively, and independent sample *t* tests were conducted (Table 6). NIC and *Z* differed significantly between the serosal-invasion and non-invasion groups (*P* < .05), whereas *ρ* did not (*P* > .05).

**Table 6:** Comparison of NIC, *ρ*, and *Z* values between groups with and without serosal invasion during the arterial phase.

Grouping	NIC	*ρ*	*Z*
Group 1	0.06 ± 0.02	−54.06 ± 11.48	7.20 ± 0.88
Group 2	0.02 ± 0.01	−55.09 ± 7.02	6.88 ± 0.15
*T* value	12.808	0.561	11.747
*P* value	.000*	.576	.000*

Group 1: with serosal invasion; group 2: without serosal invasion. Data are presented as mean ± standard deviation.

NIC = normalized iodine concentration.

* *P* < .05 was considered statistically significant.

**Figure 11.**
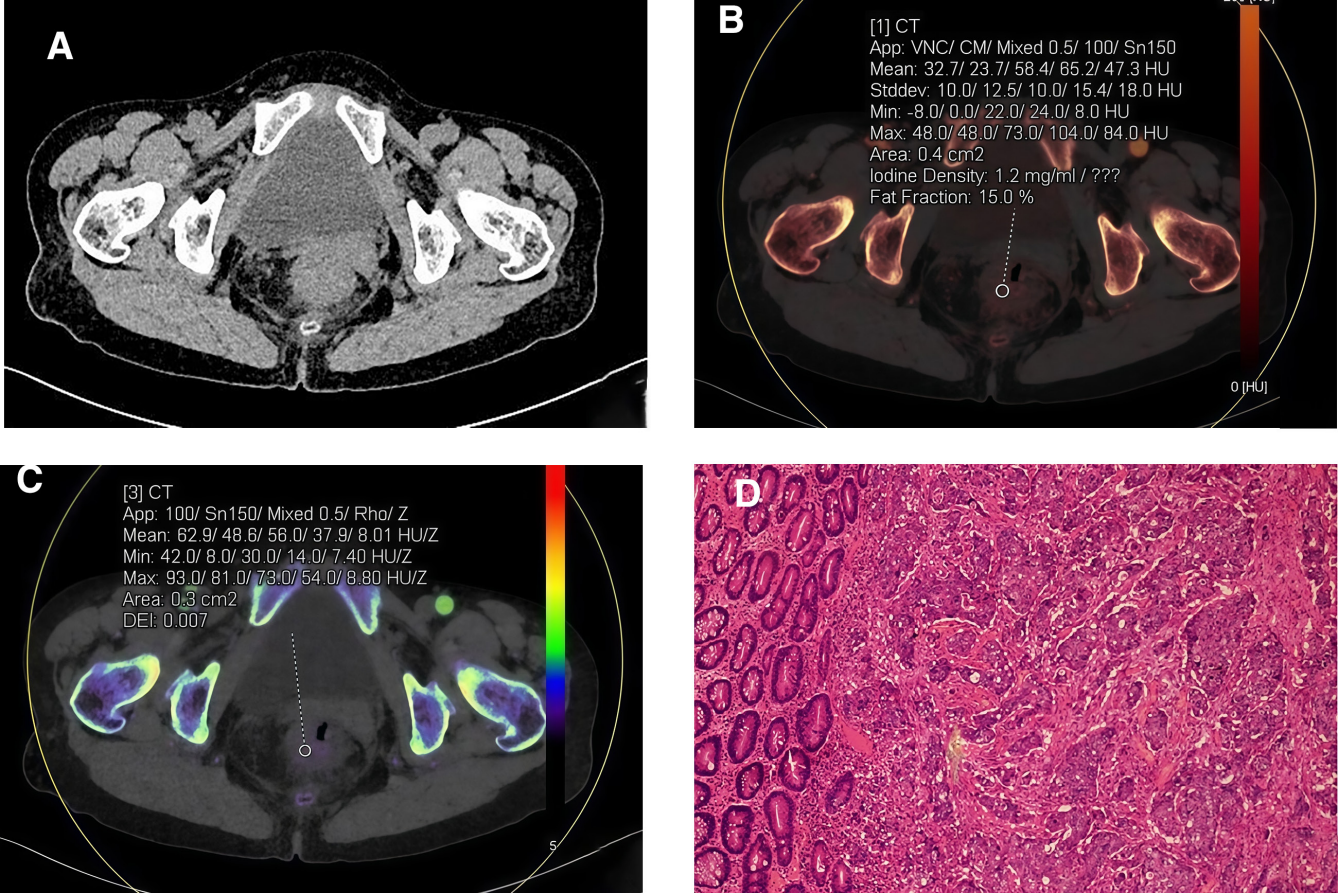
Male, 59 years old, with poorly differentiated rectal adenocarcinoma. (A) Unenhanced CT image shows irregular thickening of the rectal wall and narrowing of the lumen. (B) The iodine map from the arterial phase shows an iodine concentration of 1.2 mg/mL in arterial phase lesions. (C) The *ρ*/*Z* map from the arterial phase shows the electron density and effective atomic number diagram, indicating an electron density of 37.9 HU and an effective atomic number of 8.01 at the lesion site during the arterial phase. (D) Photomicrograph (HE ×100) depicts a poorly differentiated rectal adenocarcinoma. CT = computed tomography, VNC = virtual non-contrast.

In the venous phase, the parameters (NIC, *ρ*, and *Z*) for both groups also demonstrated normal distribution (Figs. 6–8), and independent sample *t* tests were performed (Table 7). NIC differed significantly between groups (*P* < .05), whereas *ρ* and *Z* did not (*P* > .05).

**Table 7:** Comparison of NIC, *ρ*, and *Z* values between groups with and without serosal invasion during the venous phase.

Grouping	NIC	*ρ*	*Z*
Group1	0.15 ± 0.04	−54.88 ± 11.27	7.17 ± 0.16
Group2	0.06 ± 0.03	−59.19 ± 9.10	7.12 ± 0.15
*T* value	11.389	1.916	1.349
*P* value	.000*	.058	.180

Data are presented as mean ± standard deviation.

NIC = normalized iodine concentration.

* *P* < .05 was considered statistically significant.

### 3.6. Diagnostic efficacy of parameters during arterial and venous phases for assessing serosal invasion in CRC

Parameters that demonstrated statistically significant differences were further analyzed using ROC curves.

**Figure 12.**
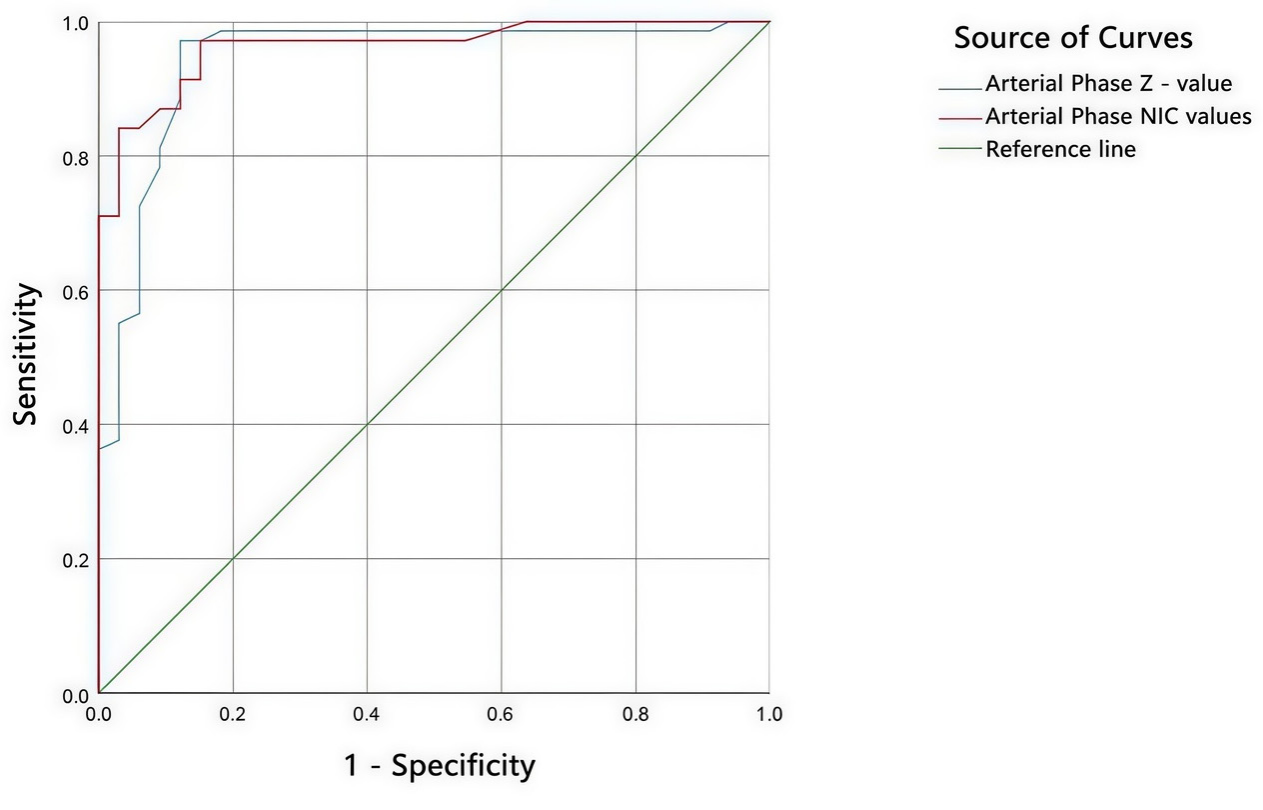
ROC curves for *Z* and NIC parameters during the arterial phase for the diagnosis of serosal invasion in CRC. CRC = colorectal cancer, NIC = normalized iodine concentration, ROC = receiver operating characteristic.

During the arterial phase, the AUCs for *Z* and NIC were 0.944 and 0.963 respectively (Fig. 12). The sensitivity for these parameters was 97.1% and 84.1%, and the specificity was 87.9% and 97.0%. Positive predictive values (PPV) were 94.4% and 95.2%, and negative predictive values (NPV) were 93.5% and 79.4%. The optimal diagnostic thresholds were 7.025 and 0.052 mg/mL, respectively (Table 8). There was no statistically significant difference in the AUC between these 2 parameters (DeLong test, *P* > .05).

**Table 8:** ROC curve analysis of *Z* and NIC for serosal invasion of CRC during the arterial phase.

Quantitative parameter	AUC	95% confidence interval		*P* value	Cutoff value	Sensitivity (Se) %	Specificity (Sp) %	PPV (%)	NPV (%)
		Upper limit	Lower limit						
*Z*	0.944	0.996	0.891	<.001	7.03	97.1	87.9	94.4	93.5
NIC	0.963	0.995	0.930	<.001	0.05 mg/mL	84.1	97.0	95.2	79.4

AUC = area under the curve, CRC = colorectal cancer, NIC = normalized iodine concentration, NPV = negative predictive values, PPV = positive predictive values, ROC = receiver operating characteristic.

**Figure 13.**
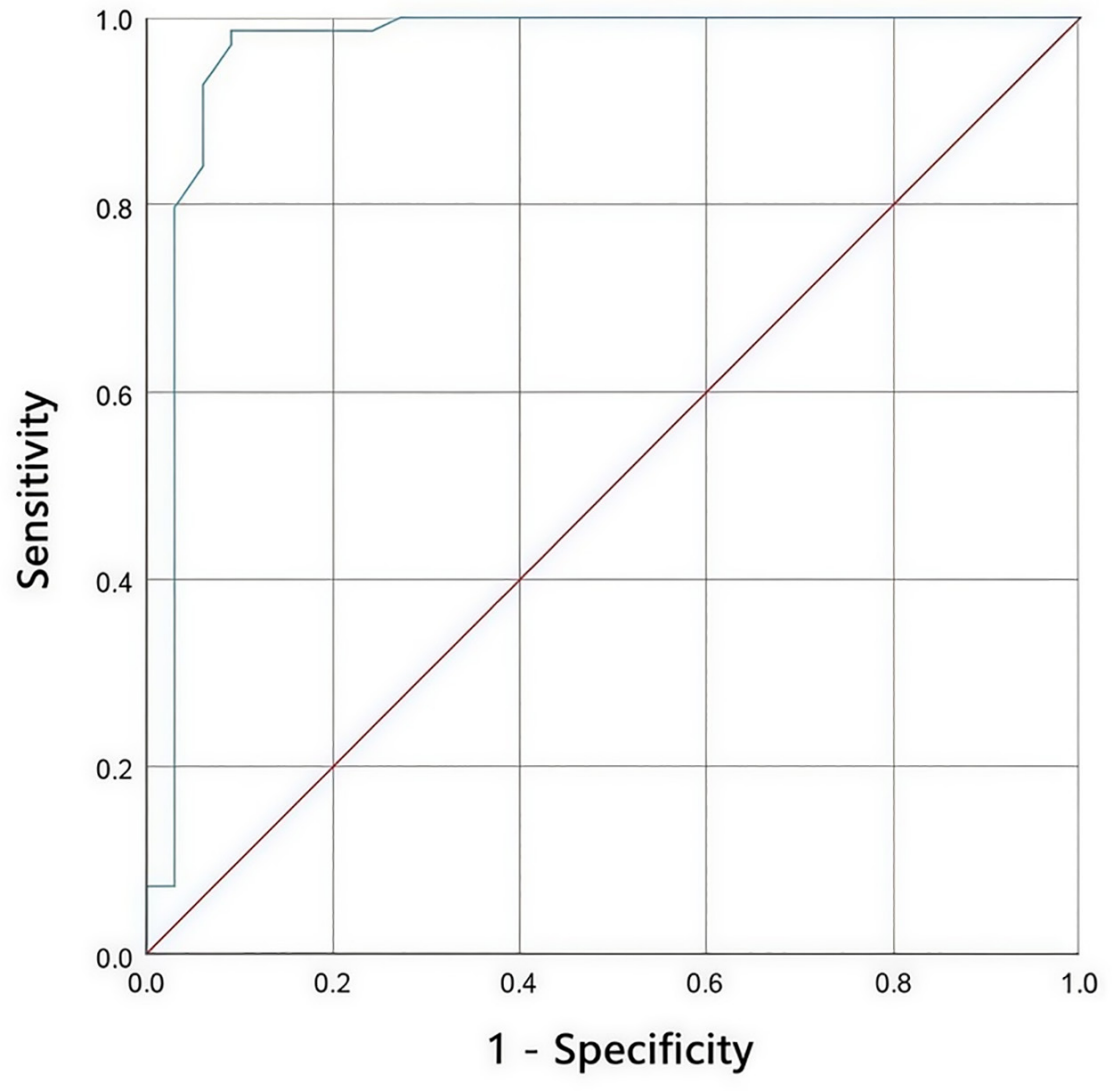
ROC curves for NIC during the venous phase for the diagnosis of serosal invasion in CRC. CRC = colorectal cancer, NIC = normalized iodine concentration, ROC = receiver operating characteristic.

In the venous phase, the AUC for NIC was 0.962 (Fig. 13), with a sensitivity of 98.6% and a specificity of 90.9%. The PPV and NPV were 95.8% and 96.8%, respectively, with an optimal diagnostic threshold of 0.093 mg/mL (Table 9).

**Table 9:** ROC curve analysis of NIC for serosal invasion of CRC during the venous phase.

Quantitative parameter	AUC	95% confidence interval		*P* value	Cutoff value	Sensitivity (Se) %	Specificity (Sp) %	PPV (%)	NPV (%)
		Upper limit	Lower limit						
NIC	0.962	0.997	0.907	<.001	0.09 mg/mL	98.6	90.9	95.8	96.8

AUC = area under the curve, CRC = colorectal cancer, NIC = normalized iodine concentration, NPV = negative predictive values, PPV = positive predictive values, ROC = receiver operating characteristic.

## 4. Discussion

The location of onset, depth of invasion, pathological grading, and TNM staging are factors that influence the surgical approach, prognosis, and survival rates in patients. The 2023 Chinese guidelines recommend different treatment pathways according to T stage, nodal status, tumor location, and pathological risk factors. Accurate preoperative assessment is, therefore, essential for selecting endoscopic treatment, segmental colectomy, neoadjuvant therapy, or postoperative chemotherapy.^[11]^

Current preoperative imaging assessments for CRC include CT, magnetic resonance imaging (MRI), endorectal ultrasound (ERUS), and positron emission tomography combined with computed tomography (PET/CT). MRI is distinguished by its superior soft tissue resolution, which facilitates clear visualization of the multilayered anatomical structure of the intestinal wall, the tumor, the surrounding adipose tissue, and the mesorectal fascia. Consequently, MRI allows for the evaluation of the circumferential resection margin.^[12]^ It is also employed in assessing the efficacy of neoadjuvant or conversion therapies.^[13,14]^ This imaging modality has been verified to provide significant diagnostic value for the T and N staging of CRC.^[15]^ Some researchers have found that by combining MRI with ERUS, the T stage can be determined more accurately. At the same time, lesions with a diameter of 1 cm can also be detected.^[16]^ CT is widely used in clinical practice for determining the clinical stage (cTNM) of CRC and non-regional lymph node metastasis and distant metastasis (cM), as well as for assessing pulmonary metastasis of CRC.^[17]^ However, both conventional CT and MDCT have limitations in distinctly imaging the various anatomical layers of the intestinal wall. They rely solely on a single parameter (CT value) and tumor morphology for analysis, which introduces certain inaccuracies in determining the T stage of the tumor.

DSCT dual-energy imaging provides both morphologic and material-specific information. By acquiring data at different energy levels, DSCT can characterize tissue composition based on energy-dependent x-ray attenuation, thereby enabling quantitative assessment using iodine maps, *ρ* maps, and *Z* maps.

Iodine mapping displays the distribution of iodinated contrast material and allows quantitative measurement of IC. This can help characterize tumor enhancement, vascularity, recurrence, and treatment response.^[18,19]^

Electron density is a measure of the number of electrons per unit pixel, which is determined by the photoelectric absorption and Compton scattering effects that occur between the base material and x-rays. In DECT, the computed *ρ* value, which is normalized against water, is expressed in HU and primarily represents the equivalent stopping power within the ROI.^[20]^

The effective atomic number indicates the atomic number of substances within an ROI, thereby determining the properties of the material. For substances within the ROI, the *Z* value provides pertinent information about their characteristics.^[19,21]^ In the case of pure substances, their *Z* value corresponds directly to their effective atomic number. For compounds or mixtures, when their x-ray attenuation coefficient aligns with that of a specific substance, the true atomic number of the substance is represented by the *Z* value within the ROI. In DSCT imaging, the *Z* value is indirectly obtained through post-processing by comparing coefficient ratios at different keV levels.^[22]^

In this study, the NIC values for the arterial and venous phases increased progressively across low, medium, and high groups. This finding may be explained by richer tumor vascularity, increased angiogenesis, and abnormal microvascular permeability in poorly differentiated tumors, resulting in greater iodine accumulation. Cao et al^[23]^ discovered that advanced or poorly differentiated CRC have elevated NIC values due to increased angiogenesis and abnormal microvascular permeability. Similarly, research by Yang^[24]^ found that in the venous phase, the NIC and *K* values for highly differentiated CRC are lower than those in poorly differentiated or undifferentiated groups, a finding consistent with our results. Some researchers have suggested^[25]^ that the NIC can also determine the pathological nature of gastric cancer lymph nodes, with metastatic lymph nodes exhibiting higher NIC values than nonmetastatic tissues (including normal and hyperplastic lymph node tissues). Peng’s research^[26]^ indicated that lung cancer lesions with lymph node metastasis have higher NIC values than those without metastasis, which may be associated with an increased microvascular density count in the metastatic lesions.

*ρ* showed no significant differences among the differentiation groups in either phase. This lack of significant difference may be related to the minimal mass and diameter of electrons, or it could be associated with the application of dual-energy technology in enhanced scanning protocols. It is believed that the *ρ* value is only applicable during plain scan imaging. Materials with a high atomic number, such as iodine or other contrast agents, may cause deviations in the measurement results.^[27]^ Atoms consist of a nucleus and extranuclear electrons, with the mass of protons or neutrons approximately 1800 times that of an electron. In the context of the entire atom, electrons can be considered negligible. Moreover, electron density values calculated using DSCT dual-energy technology are normalized relative to water. The imaging techniques in DSCT significantly reduce artifacts, thereby yielding more accurate *ρ* values.^[28,29]^

In this study, *Z* values increased as differentiation decreased, particularly in the arterial phase. Because iodine has a high atomic number, increased intratumoral iodine retention caused by rich microvasculature may increase *Z* values. Additionally, *Z* values during the venous phase generally exceeded those during the arterial phase. This may arise from the slower microcirculation within the tumor compared to the peripheral large vessels. Thus, the retention of the iodine contrast agent was prolonged, and higher *Z* values were achieved during the venous phase. Similar findings have been reported in other tumor types; for example, Li et al showed higher *Z* values in clear-cell renal cell carcinoma than in non-clear-cell renal cell carcinoma, possibly reflecting abundant tumor blood supply and stronger enhancement.^[30]^ These results suggest that the *Z* value could serve as a more direct indicator of the contrast agent content within the tumor.

In this study, the diagnostic efficacy of the NIC and *Z* values was higher in the arterial phase than in the venous phase. No significant statistical difference was found in the AUC for NIC and *Z* values between the arterial and venous phases. The positive predictive values for assessing CRC differentiation in high, medium, and low categories were relatively low in both arterial and venous phases. This might be due to the amalgamation of high and medium differentiation cases into a single group and the relatively fewer cases of low differentiation, resulting in lower positive predictive values. This suggests that both NIC and *Z* values during the arterial and venous phases possess significant diagnostic value and could serve as potential indicators for preoperatively assessing the degree of differentiation in CRC.

In this study, for serosal invasion, NIC was significantly higher in the invasion group in both phases, and arterial-phase *Z* was also significantly higher. Tumor penetration of the serosa may induce microvascular proliferation, inflammatory changes, and increased blood perfusion in perienteric fat, thereby increasing local iodine uptake and *Z* values. These results are consistent with the findings of Chen.^[31]^ Tumor microvessels are abundant, and the internal inflammatory response increases microvascular permeability, leading to higher local iodine content. In Kupeli’s^[32]^ study, it was also found that the NIC and *Z* values in the serosal invasion groups were higher than those in the non-invasion groups. The lack of a significant venous-phase *Z* difference may reflect reduced intravascular iodine concentration in perienteric fat during the venous phase and the heterogeneous composition of fat, fibrous tissue, microvessels, and inflammatory tissue.

ROC analysis showed that NIC and *Z* had high diagnostic performance for serosal invasion, with arterial-phase NIC achieving the highest AUC. NIC may be more direct than *Z* because it quantitatively reflects iodine content within the ROI, whereas *Z* represents the average atomic characteristics of heterogeneous perienteric tissues. These findings support the potential role of DSCT quantitative parameters in preoperative CRC staging.

Limitations of this study: The sample size was relatively small, particularly in the low-differentiation subgroup (n = 22). This limited sample size may have introduced sampling bias and reduced the statistical power for subgroup analyses. Multicenter studies with larger and more balanced cohorts are needed for external validation. ROI placement may be affected by observer-dependent factors despite excellent reproducibility. Some studies have shown that iodine contrast agents can cause distortion in *ρ* and *Z* values, and this study did not investigate the diagnostic value of *ρ* and *Z* during the non-contrast phase. This study was limited to the diagnostic value of a single parameter and did not explore the combined diagnostic value of multiple parameters.

## 5. Conclusion

NIC and *Z* values derived from DSCT dual-energy imaging showed high diagnostic performance for assessing CRC differentiation, whereas *ρ* showed limited value. For serosal invasion, NIC in both phases and arterial-phase *Z* demonstrated strong diagnostic performance, while the *ρ* value and venous phase *Z* value have limited value for judging serosal invasion. These parameters may serve as a noninvasive tool for preoperative risk assessment and guide individualized treatment planning.

## Acknowledgments

We thank The First Affiliated Hospital of Dali University for providing valuable data resources for this research. We are grateful to all staff who participated in data collection for their active cooperation and firm support.

## Author contributions

**Conceptualization:** Jingduo Wang, Aibo Wang.

**Data curation:** Jingduo Wang.

**Investigation:** Xiaoyuan Li.

**Resources:** Xuehua Qin.

**Software:** Chunmao Cui.

**Supervision:** Ling Liu.

**Validation:** Aibo Wang, Xuehua Qin, Chunmao Cui.

**Writing – original draft:** Jingduo Wang, Aibo Wang.

**Writing – review & editing:** Ling Liu.


