# Supplementary figures and images for: Diagnostic value of dual-source CT dual-energy technology for assessing differentiation degree and serosal invasion in colorectal cancer: A retrospective study
Source: Medicine (Baltimore). 2026 Jul 3;105(27):e49556. doi: 10.1097/MD.0000000000049556 (PMC13336986; doi:10.1097/MD.0000000000049556)

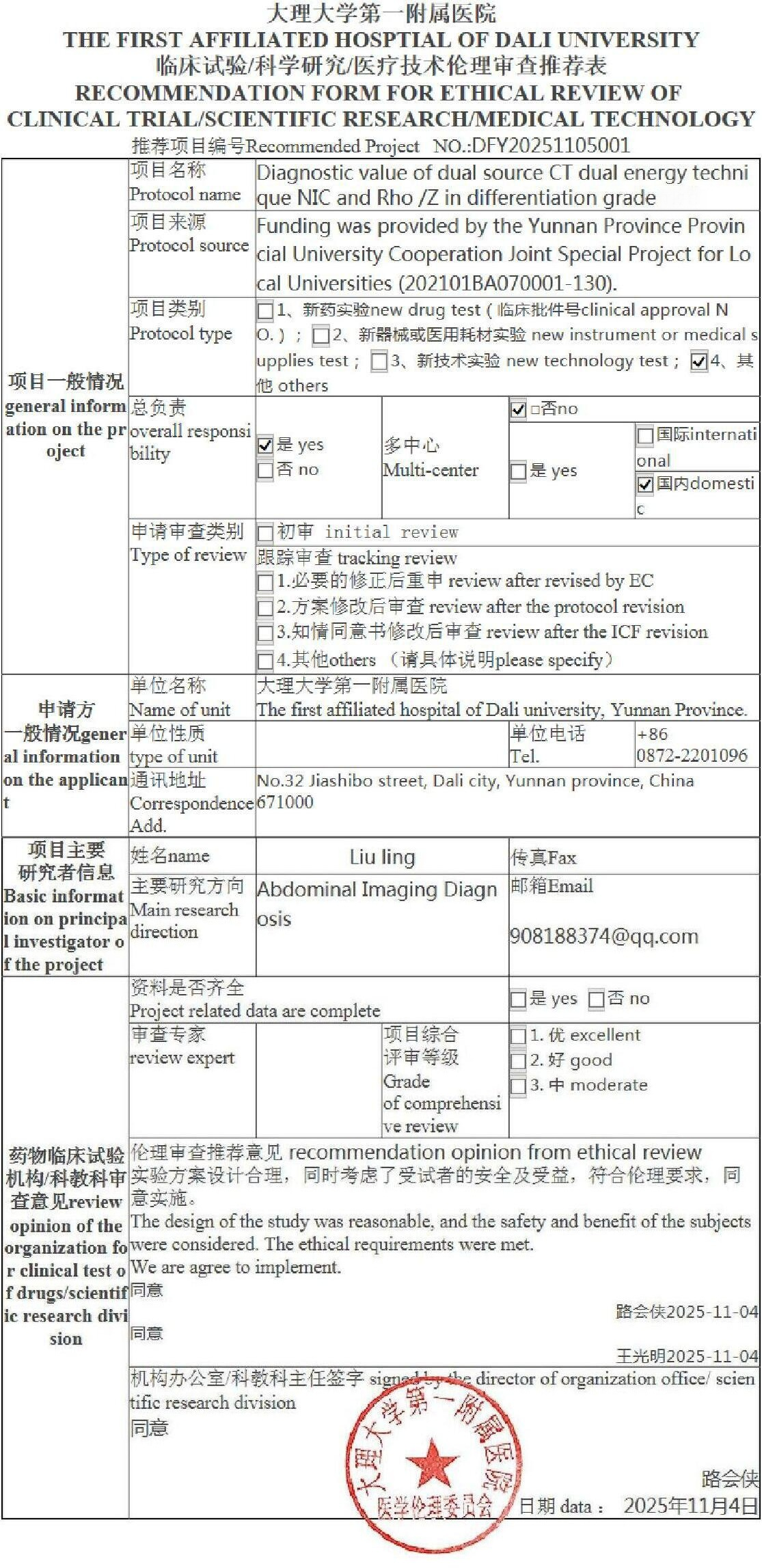

Supplement: Supplementary file 1 [file medi-105-e49556-s001.tiff]
